# *VHL* and DNA damage repair pathway alterations as potential clinical biomarkers for first-line TKIs in metastatic clear cell renal cell carcinomas

**DOI:** 10.1007/s13402-022-00691-8

**Published:** 2022-07-14

**Authors:** Jiale Zhou, Junyun Wang, Wen Kong, Jin Zhang, Xiaorong Wu, Jiwei Huang, Junhua Zheng, Yonghui Chen, Wei Zhai, Wei Xue

**Affiliations:** 1 Department of Urology, Renji Hospital, School of Medicine, Shanghai Jiao Tong University, Shanghai, 200127 China; 2 CAS Key Laboratory of Genome Sciences and Information, Beijing Institute of Genomics, Chinese Academy of Sciences/China National Center for Bioinformation, Beijing, 100101 China; 3 Department of Urology, State Key Laboratory of Oncogenes and Related Genes, Renji Hospital, Shanghai Jiao Tong University School of Medicine, Shanghai, 200127 China

**Keywords:** Clear cell renal cell carcinoma, DNA damage repair, VEGF-TKI, VHL, Predictive biomarker

## Abstract

**Purpose:**

Vascular endothelial growth factor receptor tyrosine kinase inhibitors (VEGFR-TKIs) are being used for the first-line treatment of metastatic clear cell renal cell carcinoma (mccRCC). Here, we set out to explore associations between genomic statuses, gene expression clusters and clinical outcomes of mccRCCs upon the application of VEGFR-TKIs.

**Methods:**

A retrospective study of 56 patients with mccRCC who received first-line VEGFR-TKIs and who underwent genomic profiling and whole transcriptome sequencing was conducted. Survival analysis was carried out using log-rank tests and Cox regression analyses, and Kaplan–Meier curves were plotted. Clustering was performed using the K-means method.

**Results:**

Among the 56 patients tested, 17 harbored DNA Damage and Repair (DDR) pathway alterations and 35 VHL mutations. The median progression-free survival (PFS) rates for the DDR and VHL alteration groups were 18 and 18 months, respectively, compared with 14 and 10 months for the nonmutant groups. DDR mutations, VHL mutations and co-mutations were identified as prognostic biomarkers of a longer PFS (*p* = 0.017, 0.04, 0.014). K-means clustering of expressed transcripts revealed three clusters of 40 patients: C_1, C_2 and C_3. The C_1 cluster exhibited the best PFS and objective response rate (ORR) to TKI therapy, with the highest proportion of DDR and VHL mutations. Further analysis of the tumor immune environment revealed that the C_1 cluster was enriched in activated CD8 T cells and effector CD4 T cells, whereas the C_2 cluster was enriched in eosinophils, mast cells and DC cells and, thus, in immunosuppressive cells.

**Conclusions:**

We found that patients with mccRCC harboring DDR and VHL alterations were more likely to benefit from first-line VEGF-TKI systemic therapy than patients with wild-type disease. In addition, we found that a three-cluster prognostic model based on gene expression can predict PFS and ORR, which was well-matched with activated TIL infiltration.

**Supplementary Information:**

The online version contains supplementary material available at 10.1007/s13402-022-00691-8.

## Introduction

Although the treatment of metastatic clear cell renal cell carcinoma (mccRCC) has made great progress, vascular endothelial growth factor receptor (VEGFR)-tyrosine kinase inhibitors (TKIs) remain the first-line treatment worldwide [1]. Reliable and effective biomarkers are urgently needed to improve the therapeutic response and guide targeted therapies for mccRCC. Therefore, it is considered imperative to precisely screen a specific subset of patients who could yield superior outcomes to targeted therapy.

The DDR signaling pathway represents a group of interconnected cellular signaling cascades that react in response to DNA damage [2]. Previous studies have suggested that DDR gene defects may play important roles in the progression of several malignancies. Defects in the BRCA1/2 genes in the DDR pathway are, for example, potential indicators of clinical benefit from poly-ADP-ribose polymerase inhibitor (PARPi) treatment in ovarian or breast cancers [3]. The role of DDR mutations has also been studied in urological malignancies, including prostate cancer and urothelial carcinoma [4–7]. Alterations in genes involved in DDR pathways are relatively prevalent in ccRCC, and previous studies have shown that patients with ccRCC harboring deleterious genomic alterations in DDR pathway genes might acquire superior survival benefits from immune-oncology (I/O) therapy, but not TKI-targeted therapy [8, 9]. In addition, von Hippel–Lindau (VHL) mutations are known to be the most common mutations in ccRCC, with approximately 70% of ccRCC cases harboring VHL mutations. Although somatic VHL mutation events and their impact on prognosis have been studied in a variety of studies, correlating VHL mutations with clear-cut prognostic patterns still remains a challenge and needs to be further explored [10–12]. Therefore, the association between the efficacy of TKI therapy for patients with mccRCC and the genomic status of DDR pathway genes and the *VHL* gene remains to be further elucidated.

Gene expression signatures, such as the Angio signature, have been found to be useful as biomarkers to predict improved PFS rates for TKIs in the IMmotion150 study [13]. In view of this, we conducted a retrospective biomarker study of 56 patients with mccRCC treated with TKIs to reveal associations between the genomic status of DDR pathway genes and the *VHL* gene and the response to first-line TKI therapy in mccRCC. In addition, we performed unsupervised clustering based on gene expression analysis and identified one cluster (C_1 cluster) with a longer PFS and a higher ORR among the groups of patients treated with TKIs.

## Materials and methods

### Patients and samples

A retrospective study was conducted, which included 56 patients with metastatic ccRCC treated with first-line VEGFR-TKI systemic therapy at Renji Hospital, School of Medicine, Shanghai Jiaotong University. The study was approved by the Ethics Committee of Renji Hospital, and informed consent was obtained from each patient enrolled between January 2018 and December 2020. Clinical data were collected from the PACS system and HIS system in Renji Hospital. Table 1 summarizes the clinical characteristics of the 56 patients with mccRCC studied. Median age, IMDC score, MSKCC score, and TKI agents were summarized among the overall, DDR, VHL and co-mutation groups.

**Table 1:** Clinical characteristics of 56 enrolled ccRCC patients with first-line TKIs therapy

Clinical Characteristics	Overall	DDR alteration	VHL alteration	VHL + DDR co-mutation
No. of patients	56	17	35	13
Median yrs age of at initiation of therapy(years), (range)	56(24–79)	56(41–79)	59(36–73)	56(41–73)
Gender(male)	43(76.79%)	12(70.59%)	26(74.29%)	8(61.54%)
Sarcomatoid	4(7.14%)	1(5.88%)	3(8.57%)	1(7.69%)
IMDC risk score				
Favorable	1(1.79%)	1(5.88%)	0(0%)	0(0%)
Intermediate	42(75.00%)	16(94.12%)	28(80%)	13(100%)
Poor	13(23.21%)	0(0%)	7(20%)	0(0%)
MSKCC risk score				
Favorable	1(1.79%)	1(5.88%)	0(0%)	0(0%)
Intermediate	46(82.14%)	16(94.12%)	31(88.57%)	13(100%)
Poor	9(16.07%)	0(0%)	4(11.43%)	0(0%)
VEGF**-**TKI agent				
Sunitinib	18(32.14%)	7(41.18%)	10(28.57%)	5(38.46%)
Pazopanib	6(10.71%)	0(0%)	3(8.57%)	0(0%)
Axitinib	11(19.64%)	4(23.53%)	10(28.57%)	7(53.85%)
Sorafenib	21(37.50%)	6(35.29%)	12(34.29%)	1(7.69%)

### Targeted gene sequencing and bioinformatics analysis

Targeted sequencing of all samples was performed at GloriousMed Clinical Laboratory (Shanghai) Co., Ltd. Details of the targeted sequencing and bioinformatics analyses are summarized in the supplementary materials. The targeted DDR pathway genes (67 genes) we focused on are listed in Supplementary Table 1. Somatic DDR alteration was defined as containing deleterious alterations, including frameshift insertion/deletion, nonsense, or splice site alterations and functionally validated missense mutations, in at least one of these genes.

### RNA sequencing data processing and clustering analysis by the k-means method

Raw RNA sequencing (RNA-Seq) reads were filtered by FastQC and aligned using STAR (v2.7.0 f) [14] with default parameters set to the Ensemble human genome assembly GRCh37. Gene expression levels were estimated by raw counts of transcripts per kilobase million (TPM). We performed K-means clustering based on 40 patient gene expression matrices and, finally, K = 3 was identified by testing K = 2 to K = 5. The clusters were then used as input for a PFS analysis.

### Analysis of infiltrating immune cell enrichment in the tumor microenvironment (TME) by single-sample gene set enrichment analysis (ssGSEA)

ssGSEA was used for quantifying immune infiltration and activity in tumors using gene markers reported by Wang et al. [15]. The ssGSEA method is an extension of the GSEA42 method, which works at a single-sample level rather than at a sample population. Normalized RNA-Seq data were used as input without further processing (i.e., no standardization or log transformation).

### Statistical methods

All statistical analyses were performed using R v4.1.0 (www.R-project.org; Vienna, Austria). Fisher’s exact test was used to analyze categorical variables. The Wilcoxon test was used to compare continuous variables. The clinical outcome parameters studied included progression-free survival (PFS) and best objective response rate (ORR). For PFS, Kaplan–Meier curves were plotted and compared by the log-rank test. Cox proportional hazards regression for PFS included deleterious DDR status, VHL status, sarcomatoid differentiation, IMDC risk, and MSKCC risk category at the start of therapy (favorable/intermediate/poor) as covariates for TKI analysis. The best ORR was compared between DDR mutation and groups using Fisher’s exact test. Statistical significance was set at *p* < 0.05.

## Results

### Patient characteristics

A total of 56 patients who had received first-line TKI therapy for mccRCC at Renji Hospital were analyzed in the cohort. Tumor tissues and paired blood samples or para-tumor tissues were obtained before the start of first-line systemic therapy from these patients for targeted gene sequencing and from 40 patients for whole transcriptome sequencing. Of the patients, 43 (76.79%) were male, and the median age at initiation of therapy was 56 years (range: 24–79 years). The patients’ characteristics are summarized in Table 1. The IMDC risk at the start of first-line systemic therapy was favorable in 1 (1.79%), intermediate in 42 (75.00%) and poor in 13 (23.21%) patients, whereas the MSKCC risk was favorable in 1 (1.79%), intermediate in 46 (82.14%) and poor in 9 (16.07%) patients. Among all 56 patients enrolled, 18 (32.14%) received sunitinib, 6 (10.71%) received pazopanib, 11 (19.64%) received axitinib and 21 (37.50%) received sorafenib as first-line systemic therapy. All tissues were obtained from surgical resection or biopsy of the primary tumor.

### Overall design and DDR mutation landscape in mccRCC

**Fig. 1:**
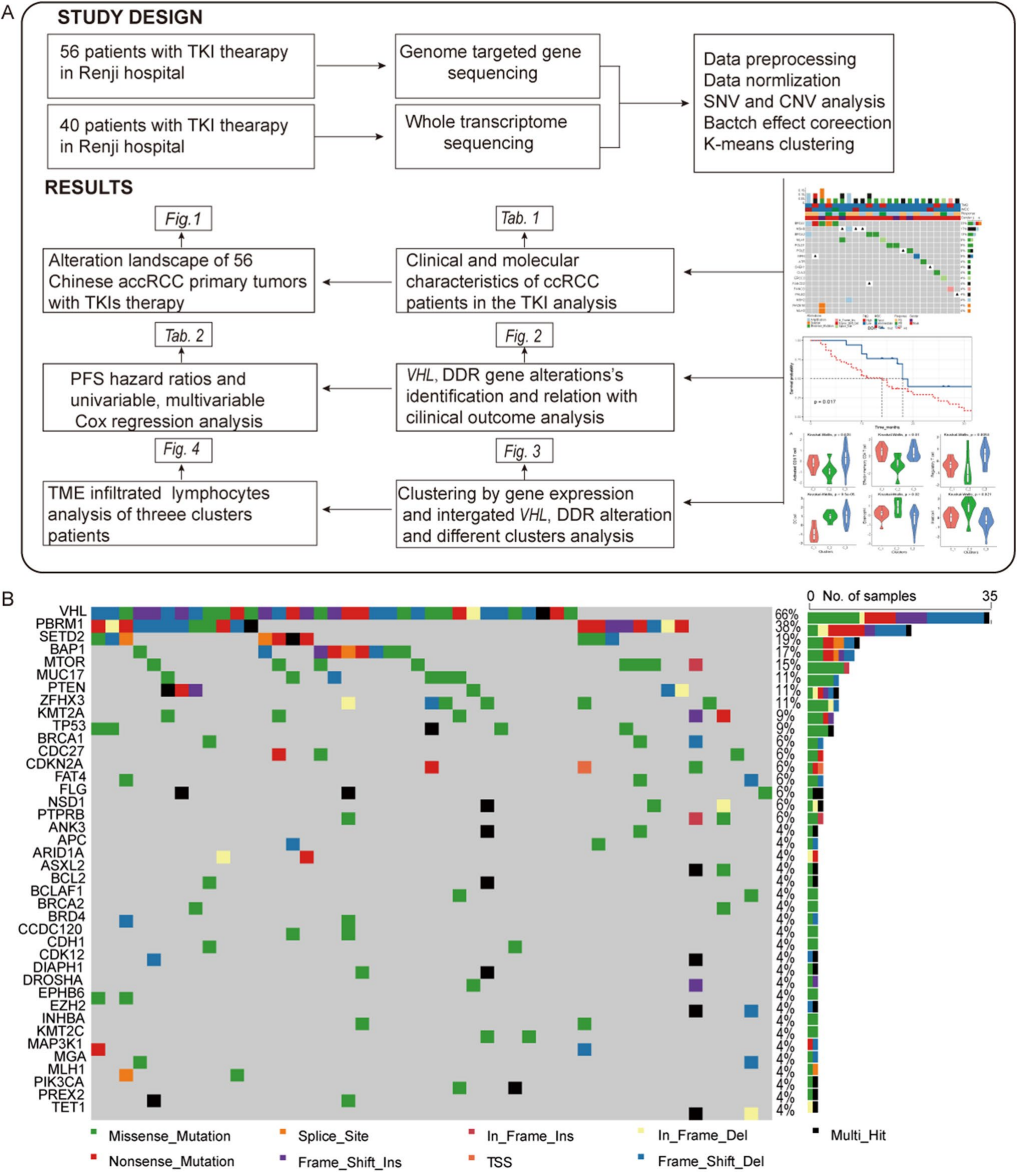
Flow chart of genomic and transcriptomic analyses and overview of the Renji-mccRCC cohort with first-line VEGF-TKI therapy and its somatic mutation landscape. (**A)** All 56 patients underwent targeted genome sequencing, and 40 were analyzed by whole transcriptome sequencing. The overall study design and main results are shown in the flow chart. (**B)** The top 40 genes of the somatic mutation oncoplot are shown. *VHL* mutations were found in 35 (66%) of the patients, followed by *PBRM1* mutations in 20 (38%) patients and *SETD2* mutations in 10 (19%) patients

The overall design of this research is illustrated in Fig. 1A, and the somatic mutation landscape in mccRCC is shown in Fig. 1B. Among all 56 patients’ somatic mutations, *VHL* mutations were found in 35 (55%) of the patients, followed by *PBRM1* mutations in 21 (38%) patients and *SETD2* mutations in 10 (19%) patients.

**Fig. 2:**
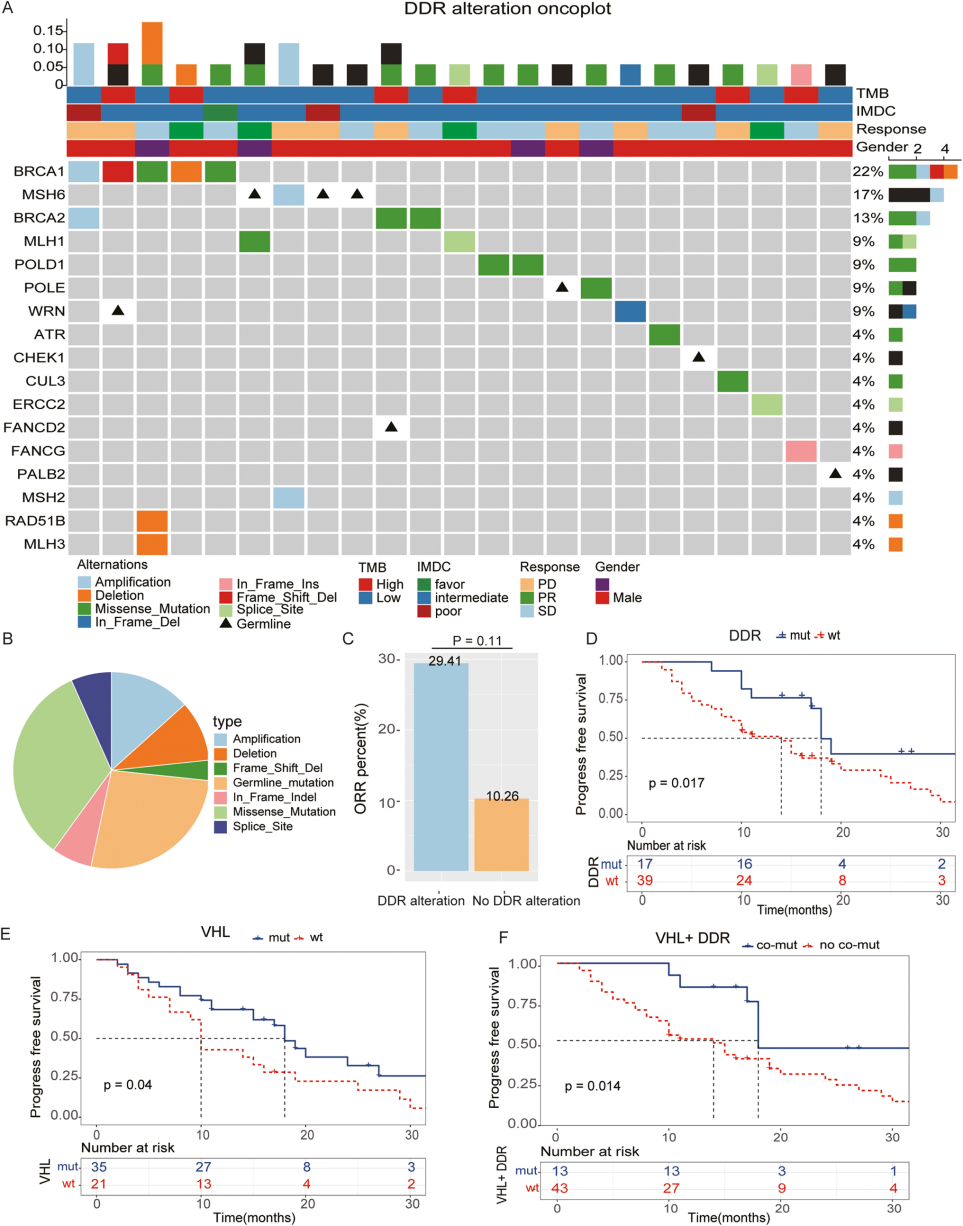
Somatic *VHL* and DDR alterations are associated with treatment outcome and PFS in 56 patients with first-line VEGF-TKI therapy. (**A)** DDR genomic alteration oncoplot of 56 patients. A total of 30 DDR gene alterations were detected in 17 patients. (**B)** Distribution of deleterious somatic and germline alterations and CNVs (amplifications and deletions) in patients with mccRcc. (**C)** Comparison of the ORR percentages of the DDR pathway mutant group and the nonmutant group. DDR mutant patients tended to show a better ORR rate than the nonmutant patients. (**D**-**F)** Kaplan–Meier plots were used to estimate PFS according to DDR alteration, *VHL* mutation and co-mutation status. Patients harboring *VHL* mutations, DDR alterations or *VHL* and DDR co-mutations showed a longer PFS than the corresponding nonmutation patients in first-line TKI treatment (log-rank *p* = 0.04, 0.017, 0.011)

Overall, 30 DDR gene alterations were detected in 17 patients in this cohort, with at least one alteration per patient (range 1 to 3) (Fig. 2A). The most common mutation type was missense mutation (*n* = 10, 33.3%), followed by amplification (*n* = 4, 13.3%) and deletion (*n* = 3, 10%) (Fig. 2B). The most frequent DDR gene alteration was observed in BRCA1 (*n* = 5), followed by MSH6 (*n* = 4) and BRCA2 (*n* = 3). Eight patients harbored pathogenic germline alterations, including MSH6 (*n* = 3), POLE (*n* = 1), WRN (*n* = 1), CHEK1 (*n* = 1), FANCD2 (*n* = 1) and PALB2 (*n* = 1) (Fig. 2A).

### ORR responses and PFS outcomes to TKI-targeted therapy by genomic status of VHL and DDR pathway genes

We evaluated the proportion of ORR responses in the 56 patients with ccRCC who received first-line TKI therapy. The ORR response was observed more frequently in the DDR alteration group than in the non-DDR alteration group (29.41% vs. 10.26%, *p* = 0.11) (Fig. 2C). Evaluation of clinical outcomes in somatic alteration subgroups revealed that DDR mutations conferred a better prognosis.

With a median follow-up duration of 16.5 months (range: 2 to 53 months), 41 events (disease progression) were recorded among the 56 patients, and 33 patients died of the disease. The median PFS for the current cohort was 11.0 (95% CI: 11.27 ~ 15.70) months. The median PFS for patients with DDR pathway alterations, *VHL* alterations, and no alterations was 18.0 versus 14.0 months and 18.0 versus 10.0 months, respectively.

Through Kaplan–Meier analysis of PFS, we found that patients in either the VHL mutation, DDR alteration or co-mutation groups showed longer PFS rates than those in the corresponding nonmutation groups after first-line TKI systemic treatment (log-rank *p* = 0.04, 0.017, 0.011) (Fig. 2D, E, F). These data suggest that VHL and DDR alterations confer a better prognosis compared with non-altered tumors.

### Prognostic factor evaluation of patients with mccRCC treated with TKI-targeted therapy

In univariable analysis, poor IMDC risk, poor MSKCC risk and the presence of sarcomatoid differentiation in tumors, as well as VHL and DDR gene alterations, were also found to serve as risk factors for a poor PFS after first-line TKI systemic therapy. In multivariable Cox analysis, poor IMDC risk, sarcomatoid differentiation and VHL wild type served as independent risk factors for a poor PFS in TKI therapy (Table 2).

**Table 2:** Univariable and multivariable Cox regression models to predict PFS

Prognostic Factors Univariable		Univariable Multivariate analysis		Multivariate analysis
	HR (95% CI)	*p*-value	HR (95% CI)	*p*-value
Gender.Male	0.966(0.52–1.793)	0.912		
Age.Old	0.966(0.415–2.248)	0.935		
IMDC.Poor	2.54(1.247–5.172)	0.010	3.97(1.08–14.62)	0.038
MSKCC.Poor	2.228(1.012–4.906)	0.047	0.57(0.14–2.24)	0.418
Sarcomatoid	3.089(1.06–8.998)	0.039	5.03(1.6–15.76)	0.006
VHL.mut	0.526(0.284–0.975)	0.041	0.47(0.25–0.91)	0.024
DDR.mut	0.405(0.186–0.882)	0.023	0.57(0.25–1.32)	0.189

### Identification of three molecular ccRCC subtypes and subtypes associated with differential clinical outcomes after TKI therapy

**Fig. 3:**
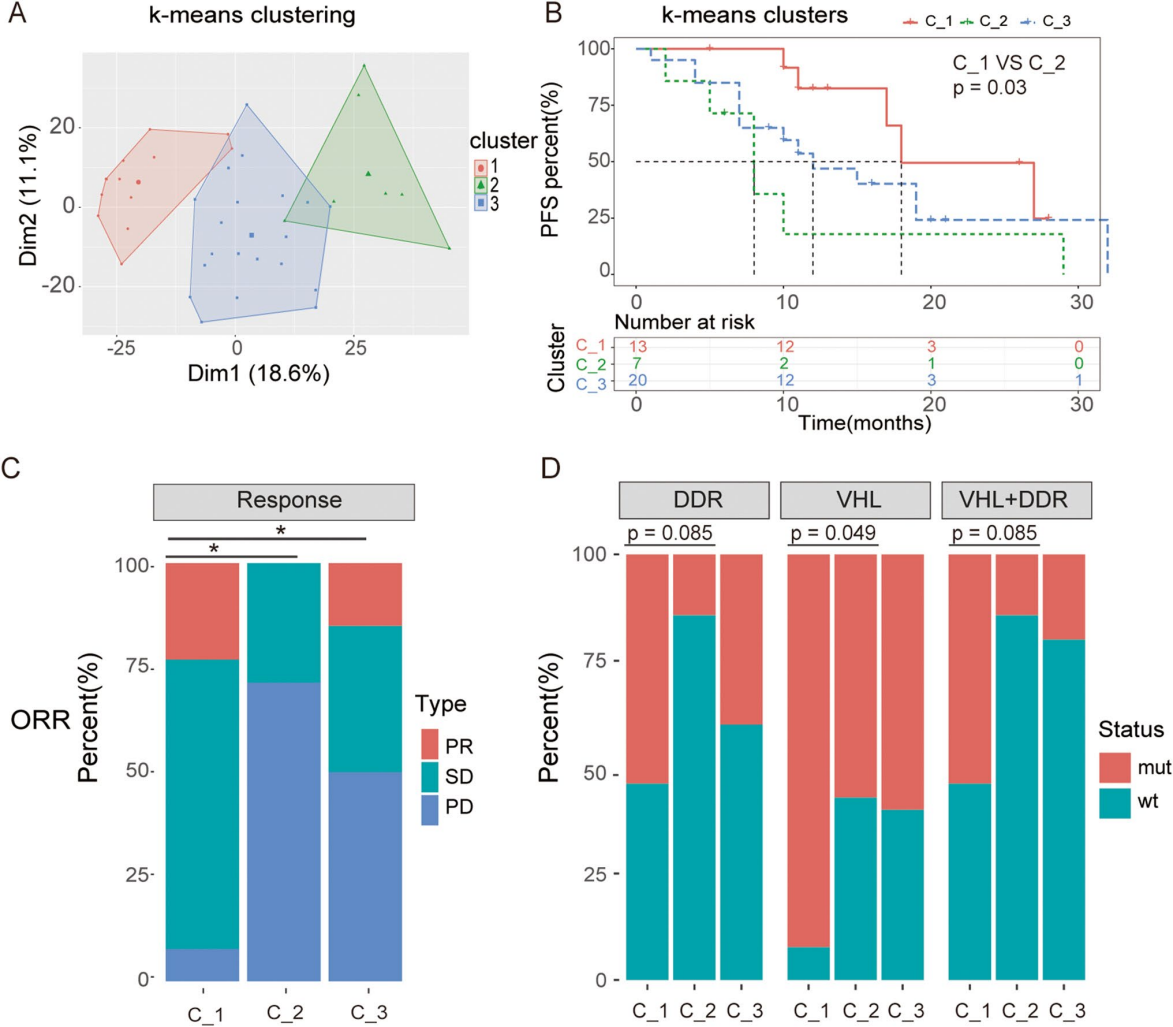
Transcriptional clustering identifies three molecular subtypes of mccRCC and subtypes associated with differential clinical outcomes after TKI therapy. (**A)** 40 patients with mccRCC were divided into three different clusters, C-1 (TIL-abundant), C-2 (immune-inhibited) and C-3 (TIL-intermediate) by K-means clustering. (**B)** Kaplan–Meier survival analysis showing that patients in the C_1 cluster exhibited longer PFS rates than those in the C_2 and C_3 clusters. The log rank *p* value between C_1 and C_2 was 0.03, whereas the *p* value between C_1 and C_3 was 0.13. (**C)** Stacked bar diagram showing the ORR percentages of the three subgroups. Fisher’s exact test was used to compare differences. C_1 versus C_2, *p* = 0.0187; C_1 versus C_3, *p* = 0.0301, *, *p* < 0.05. (**D)** Different subtypes were associated with distinct proportions of somatic *VHL* mutations, DDR alterations and co-mutations. The C_1 subgroup harbored a higher percentage of genomic *VHL* and DDR mutations than the C_2 and C_3 subgroups

To explore more predictive biomarkers based on gene expression signatures, whole transcriptome sequencing was conducted in 40 patients with available tissue samples. Using K-means clustering methods, the 40 patients with mccRCC were divided into three different clusters: C_1, C_2 and C_3 (Fig. 3A). We subsequently evaluated the clinical outcomes of TKI treatment in each cluster and found that C_1 subgroup patients exhibited a longer PFS than those in the C_2 and C_3 treatment groups (C_1 vs. C_2, *p* = 0.03, C_1 vs. C_3, *p* = 0.13) (Fig. 3B). In addition, we found that the C_1 subgroup exhibited a better ORR rate to first-line TKI therapy than the C_2 and C_3 subgroups, and this result was statistically significant (C_1 vs. C_2 *p* = 0.0187, C-1 vs. C-3 *p* = 0.0301) (Fig. 3C). In other words, we achieved population stratification at the transcriptional level and identified one subtype (C_1 cluster) that was more likely to benefit from targeted therapy by our model.

### Somatic *VHL* and DDR alterations are associated with different transcriptional subtypes

To further explore the correlation of VHL and DDR alterations with the different subgroups, we complemented transcriptional profiling with evaluation of somatic alterations in tumors from 40 patients. Consistent with the previous variation analysis, we found that the C_1 cluster with a better PFS tended to harbor more *VHL* mutations than the C_2 cluster (*p* = 0.049). This trend of a longer PFS in VHL mutation patients was also observed in the DDR mutant and co-mutant subgroups, but did not reach statistical significance (*p* = 0.085, 0.085). Overall, we found that VHL and DDR genes with frequent deleterious alterations are associated with good clinical outcomes after TKI therapy, suggesting that targeted somatic mutation profiling in mccRCC could help to guide clinical treatment selection.

### Transcriptional analysis identifies mccRCC subtypes with distinct tumor immune microenvironments

**Fig. 4:**
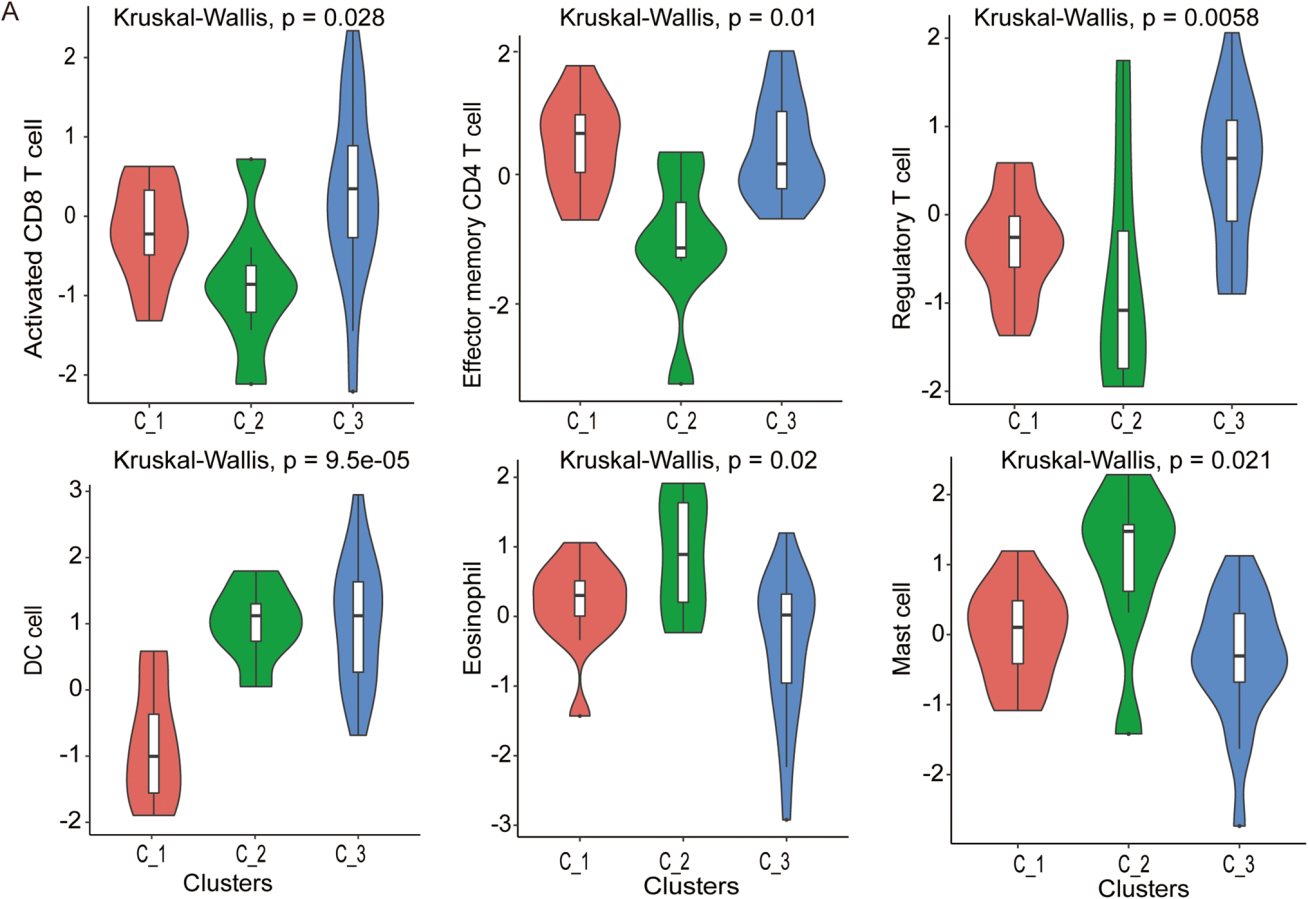
Different subtypes exhibit unique characteristics of the tumor immune microenvironment. The C_1 subgroup was abundant in activated TILs, including activated CD8 + T cells and effector memory CD8 + T cells, whereas the C_2 group was abundant in suppressed TILs, such as eosinophils, mast cells and DCs. The C_3 subtype was abundant in Treg cells

To further reveal the relationship between the tumor immune microenvironment and the impact on different subgroups, we quantified immune infiltration in tumors of the three clusters using ssGSEA methods. We found that C_1 is abundant in activated tumor infiltrating lymphocytes (TILs), including activated CD8 + T cells, effector memory CD8 + T cells and NK cells, and can be classified as immune-activated cluster. In contrast, we found that C_2 showed more suppressed TILs, such as eosinophils, mast cells and DCs and, thus, can be considered as immunosuppressed cluster. The C_3 subtype was abundant in Treg cells and inferred not to be susceptible to ICIs. The status of TILs in the three clusters strongly coincides with the PFS prognosis. The abundance of significantly different TILs in the three clusters is illustrated in Fig. 4, and the overall profile of the 28 TILs is shown in Fig. S1. The tumor immune microenvironment (TIME) suggests that the abundance of TILs may also serve as a potential predictor of the response to TKI therapy.

## Discussion

We performed a retrospective biomarker study to assess associations between the genomic status of the *VHL* gene and DDR pathway genes, and the sensitivity to first-line TKI therapy. Targeted sequencing was conducted in a cohort of 56 patients with metastatic ccRCC, and whole transcriptome sequencing was conducted in 40 patients from the same cohort. DDR pathway and *VHL* gene mutations were identified as prognostic biomarkers for a longer PFS after TKI therapy. Through transcriptome sequencing, a 3-cluster model was constructed based on the presence of tumor-infiltrating lymphocytes and the concomitant clinical response to first-line TKI therapy.

The clinical significance of VHL in the prognosis and treatment of ccRCC is widely recognized [[16]]. However, the clinical value of DDR genes in ccRCC and its systemic treatment has rarely been addressed. DDR mutations have been found to be correlated with clinical benefit from PD-1/PD-L1 therapy, but their clinical significance for TKI therapy has remained unclear. In the current study, 30 DDR gene mutations were identified in 17 patients. Through Kaplan–Meier analysis, patients with DDR mutations were found to be more likely to benefit from first-line TKI therapy and to exhibit longer PFS rates than patients with wild-type DDR genes, but no significant difference was found in OS. These results differ from those previously reported by Ged et al. [8]. According to this report, no differences were found in either OS or PFS between patients with and without DDR mutations in a subset of 118 patients in their TKI cohort. This discrepancy may be explained by the fact that the sizes of the panels and the numbers of DDR genes tested were different. In our panel we tested more than 640 key genes and 67 DDR genes, while Ged’s panel consisted of more than 400 genes and 34 DDR genes. Also, the baseline characteristics of the patients between the two study cohorts were different. First, the IMDC risk score at starting VEGF-TKI therapy was quite different in the two cohorts: the proportion of favorable risk levels in Ged’s study (*n* = 32, 29%) was larger than that in our study (*n* = 1, 1.79%). Second, the composition of VEGF-TKI agents in both groups differed from each other. In Ged’s study, the patients mainly received sunitinib (*n* = 73, 62%) or pazopanib (*n* = 42, 36%) as first-line systemic therapy, whereas the choice for first-line systemic therapy varied in our study. Sorafenib (*n* = 21, 37.5%) was most used in the current cohort, followed by sunitinib (*n* = 18, 32.14%), axitinib (*n* = 11, 19.64%) and pazopanib (*n* = 6, 10.71%). This may explain why diversity may exist in the clinical responses to different VEGF-TKI agents in patients with DDR mutations. Unfortunately, due to the limited sample size in this study, we failed to provide clinical-significant outcomes about which agent is most beneficial for patients with DDR mutations.

For early diagnosis and better treatment of patients with high risk RCC, increasing attention has recently been paid to the identification of prognostic parameters for patients with mRCC and to the development of prognostic models [17]. However, by reviewing previous prognostic models, we found that these models mainly predict prognosis and survival of all patients with RCC, whereas only few models focus on the clinical response of patients with mRCC receiving VEGF-TKIs as first-line treatment. To date, among all existing prognostic models, two well-designed models, the MSKCC (Memorial Sloan Kettering Cancer Center) and IMDC (International Metastatic Renal Cell Carcinoma Database Consortium) models have been widely used [18, 19]. However, both models are based on clinical parameters, so the two models share the same caveat of lacking genomic heterogeneity. Therefore, several problems have arisen during the clinical application of the two models. For example, a lack of balance in patient distribution in the three risk groups has long been recognized. Approximately 50% of the patients fall into an intermediate risk group, which was also noted in both Ged’s study and our study [20]. With urologic studies coming into the genomic era, multi-omics studies have provided new methods for the development of prognostic models [21, 22]. These include a model described by Ricketts et al. [21] resulting from proteogenomic characterization of 103 treatment-naïve ccRCC patient samples showing tumor-specific alterations at the proteomic level and on which a revised subtyping scheme was based. Comprehensive genomic and phenotypic analyses were conducted on 843 RCC samples from different patients and revealed genomic, pathway alteration, mRNA signature and metabolic alterations. However, by overviewing the existing genomic models, few were found to be directly related to metastatic ccRCC and prognosis. Here, we constructed a three-group model based on differential expression patterns and the presence of TILs, which can predict PFS and clinical response in patients with mRCC treated with VEGF-TKIs. In a previous study, a positive correlation between the abundance of TILs and a favorable prognosis was found in RCC. But, there seems to be controversy about the role of different TILs in RCC [23]. In our study, the C_1 cluster represented the TIL-activated subtype, including a higher abundance of activated CD8 + T cells, effector memory CD4 + cells, and fewer DC cells, eosinophils and mast cells that suppress immune cells. The TIL-activated group was found to be directly correlated with good clinical outcomes after initiating first-line VEGF-TKI therapy.

Nevertheless, our study has several limitations. All patients in the retrospective cohort were treated in a single center, and as mentioned before, the limited sample size did not allow us to further analyze the significance of single genes and subgroups.

In conclusion, we found that patients with mccRCC with *VHL* and DDR gene alterations are more likely to benefit from first-line VEGF-TKI systemic therapy than patients without such mutant genes. In addition, a three-group TIL-based prognostic model was established by k-means analysis. We found that the TIL-activated group was correlated with better PFS and ORR responses. Further large-cohort multicenter studies are needed before clinical application.

## Supplementary Information

Below is the link to the electronic supplementary material.

Supplementary file1 (TIF 14087 KB)

Supplementary file2 (DOCX 18 KB)

Supplementary file3 (XLSX 17 KB)

## Data Availability

The supporting data will be available upon reasonable request.
