# Supplementary figures and images for: VHL and DNA damage repair pathway alterations as potential clinical biomarkers for first-line TKIs in metastatic clear cell renal cell carcinomas
Source: Cell Oncol (Dordr). 2022 Jul 14;45(4):677–87. doi: 10.1007/s13402-022-00691-8 (PMC9424144; doi:10.1007/s13402-022-00691-8)

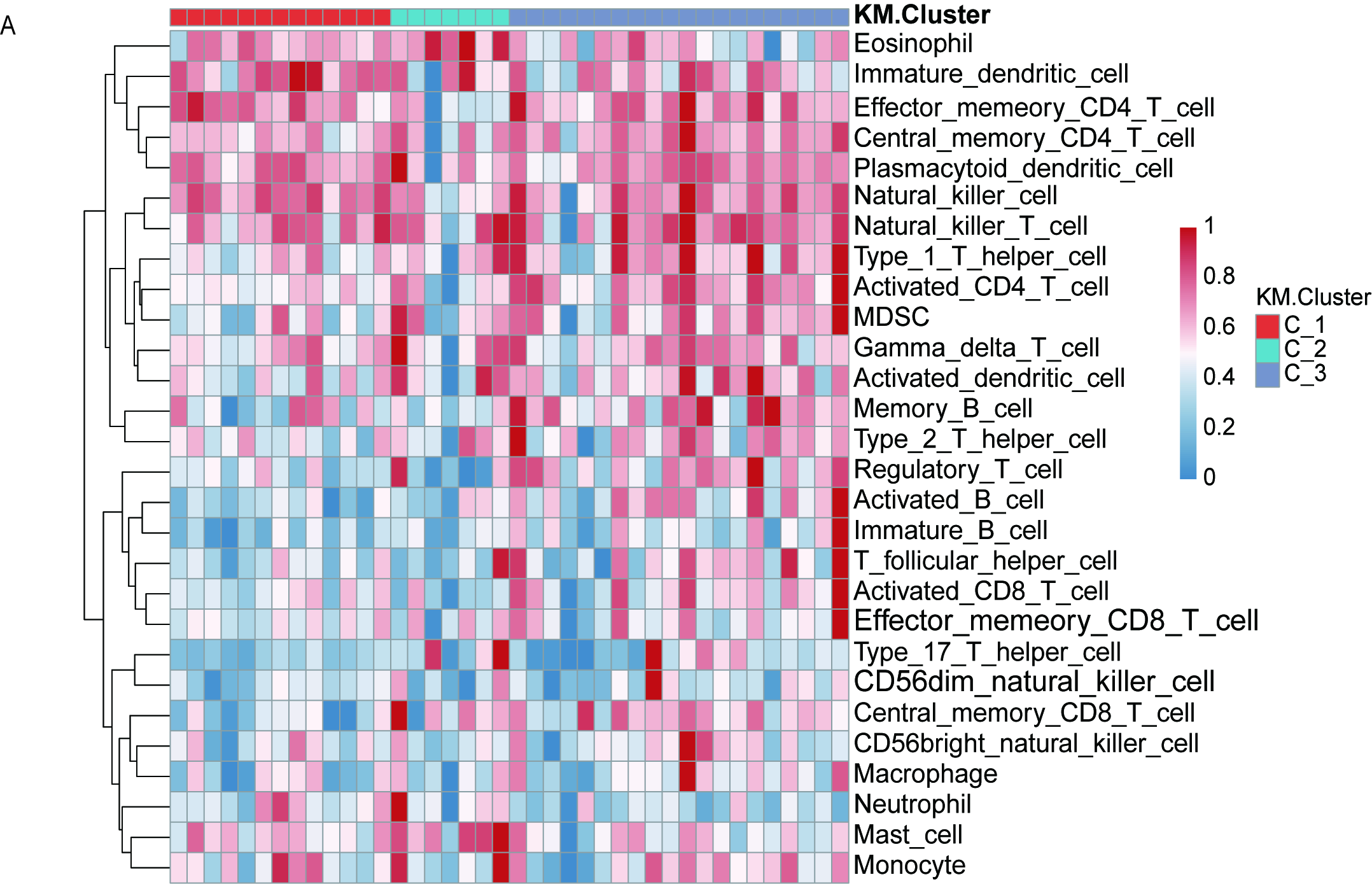

Supplement: Supplementary file 1 — Supplementary file1 (TIF 14087 KB) [file 13402_2022_691_MOESM1_ESM.tif]
