# Supplementary material for: VHL and DNA damage repair pathway alterations as potential clinical biomarkers for first-line TKIs in metastatic clear cell renal cell carcinomas
Source: Cell Oncol (Dordr). 2022 Jul 14;45(4):677–87. doi: 10.1007/s13402-022-00691-8 (PMC9424144; doi:10.1007/s13402-022-00691-8)
Supplement: Supplementary file 2 — Supplementary file2 (DOCX 18 KB) [file 13402_2022_691_MOESM2_ESM.docx]

**Supplementary Methods**

1. Targeted Gene Sequencing

Tumor DNA was extracted from 5 to 10 formalin-fixed, paraffin-embedded (FFPE) sections (5 um thick) using a QIAamp DNA FFPE Tissue Kit (Qiagen), and Blood Genomic DNA Mini Kit (Cwbiotech, Beijing, China) was used to extract Genomic DNA (gDNA) from white blood cells (WBCs). Twenty to 100 ng of cfDNA, 200 to 500 ng of FFPE DNA, or 500 ng of gDNA were then used for library preparation and quantification, guided by the protocols of the KAPA Hyper Prep kit (Roche, Basel, Switzerland). Final libraries were sequenced on Illumina Nextseq500 (PE 75) or Novoseq 6000 (PE 150) sequencers (Illumina, San Diego, CA, USA).

2. Quality control and variant calling

Trimmomatic^1^ was used to trim the sequencing adapters from the raw data. The reads were aligned with the human reference genome (hg19) using BWA^2^. Duplicated reads were removed using Picard (http://broadinstitute.github.io/picard/). Mapped reads were realigned to the genome using Genome Analysis Tool Kit ^3^. Somatic mutations and germline mutations were called using Mutect2 and GATK’s Haplotype Caller ^3^ with a paired workflow, respectively.

Variants were then annotated using ANNOVAR ^4^ and in-house developed code. An in-house script was used to verify the human identity concordance of paired samples. Somatic copy number alterations were also detected using GATK ^3^.

3. Germline and somatic variant filtering

With a threshold of minimum coverage of 50 × and an allele frequency of over 30%, germline variants called by GATK on WBC samples were filtered initially. Then, variants not on coding regions and synonymous mutations annotated using ANNOVAR [4] were filtered out. Furthermore, variants with a population minor allele frequency over 0.1% (annotated using the ExAC database) were considered less functional. Functional filtering removed variants located in non-coding regions and synonymous mutations were removed for downstream analysis. A log2 ratio more than 0.6 was considered a copy gain. A log2 ratio less than 0.7 was considered a copy loss.

References for supplementary methods:

1. Bolger, A. M., Lohse, M., Usadel, B.: Trimmomatic: a flexible trimmer for Illumina sequence data. Bioinformatics, 30: 2114, 2014

2. Li, H., Durbin, R.: Fast and accurate short read alignment with Burrows-Wheeler transform. Bioinformatics, 25: 1754, 2009

3. McKenna, A., Hanna, M., Banks, E. et al.: The Genome Analysis Toolkit: a MapReduce framework for analyzing next-generation DNA sequencing data. Genome Res, 20: 1297, 2010

4. Wang, K., Li, M., Hakonarson, H.: ANNOVAR: functional annotation of genetic variants from high-throughput sequencing data. Nucleic Acids Res, 38: e164, 2010
